# Successful treatment of dyshidrotic palmoplantar eczema with ultraviolet A1 light‐emitting diodes

**DOI:** 10.1111/1346-8138.15402

**Published:** 2020-05-22

**Authors:** Kyoko Ikumi, Tomohiko Kio, Kan Torii, Hideyuki Masuda, Akimichi Morita

**Affiliations:** ^1^ Department of Geriatric and Environmental Dermatology Nagoya City University Graduate School of Medical Sciences Nagoya Japan; ^2^ R&D Group Biomedical Division USHIO INC Tokyo Japan

**Keywords:** Dyshidrotic Eczema Area and Severity Index, dyshidrotic palmoplantar eczema, phototherapy, pigmentation, ultraviolet A1 light‐emitting diodes

Dear Editor,

Ultraviolet (UV)‐A1 was first reported to be an effective treatment for atopic dermatitis in the 1990s,^1^ and has since been widely applied for atopic dermatitis,^2^ cutaneous T‐cell lymphoma, localized and systemic scleroderma,^3^ and dyshidrotic palmoplantar eczema.^4^ UVA1 irradiation is traditionally produced by methyl halogen lamps emitting 340–400‐nm wavelengths with three filters. A major drawback of these lamps, however, is that they consume large amounts of electricity and generate unnecessary heat.

To address this problem, we developed a new treatment device (based on the TheraBeam^®^ UV‐N; Ushio, Tokyo, Japan) incorporating light‐emitting diodes (LED) instead of lamps. This device emits a peak wavelength of 365‐nm UVA1 and utilizes a 355‐nm or shorter wavelength cut‐off filter to theoretically reduce unnecessary immediate pigment darkening (IPD). The peak wavelength of the IPD action spectrum is approximately 340 nm. Therefore, IPD can be suppressed by installing a filter (H. Masuda *et al.*, 2020, unpubl. obs.). The light source unit comprises 64 LED packages with a typical output power of 500 mW and an irradiance of 84 mW/cm^2^ at body distance, as measured with an IL 1700 photometer (International Light, Newburyport, MA, USA).

We investigated the efficacy of UVA1 LED treatment for patients with dyshidrotic palmoplantar eczema. The study was a prospective interventional study approved by the institutional review board of Nagoya City University Graduate School of Medical Sciences (approval no. 46‐18‐0014). The treatment protocol was a UVA1 dose of 30 or 60 J/cm^2^ once a week for 5 weeks. The clinical course of the patients was followed at regular intervals. The clinical scores were calculated using the modified Dyshidrotic Eczema Area and Severity Index (DASI) score: modified DASI score = (Pv + Pe + Ps) * Pa, where “P” indicates points (0–3), “v” vesicles, “e” erythema, “s” desquamation and “a” area.^5^

Ten patients were recruited for this study and treated with UVA1 LED. Representative cases are shown in Figure 1(c). The clinical efficacy and courses of the patients are shown in Figure 1(b,d). Of the 10 patients, six completed the UVA1 LED treatment. Treatment was terminated in four patients because three achieved complete remission prior to completing the 5‐week treatment course and one patient experienced worsening eruptions (case 4, Fig. 1c, lower left). In cases 1 and 7, a single UVA1 LED treatment completely alleviated the disease (Fig. 1c, upper left). In case 2, UVA1 LED treatment allowed for decreasing the dose of cyclosporin (Fig. 1c upper right). In nine patients, UVA1 LED irradiation did not increase itching or warmth at the irradiated sites. The treatment decreased disease severity (Fig. 1d). UVA1 LED treatment significantly improved the DASI scores (Wilcoxon matched‐pairs signed rank test, *P* < 0.01). The modified DASI scores improved each week. UVA1 LED treatment also appeared to shorten the acute vesicular stage (Fig. 1d).

**Figure 1:**
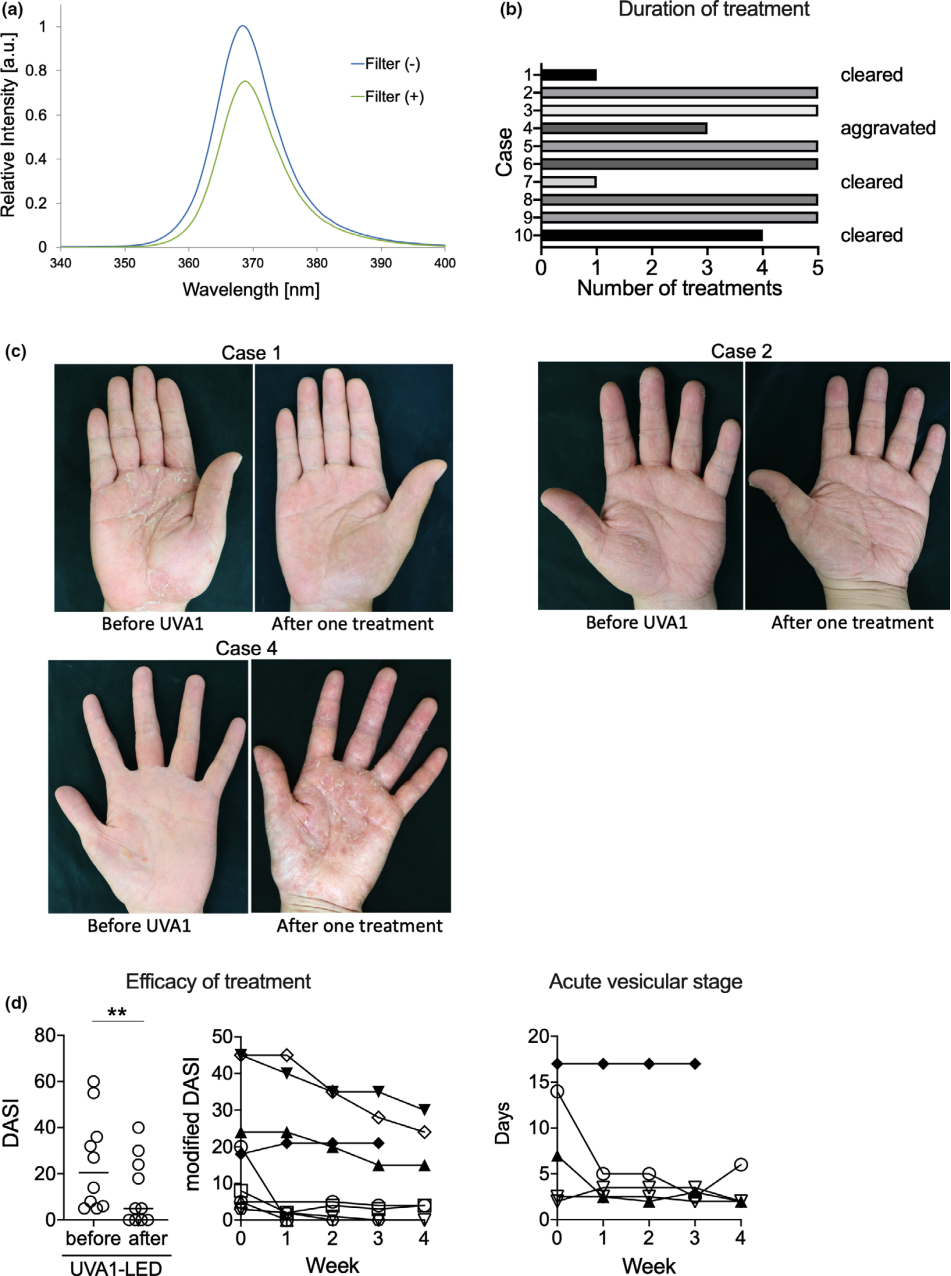
Ultraviolet (UV)A1 light‐emitting diode (LED) treatment with a cut‐off filter alleviated dyshidrotic palmoplantar eczema. (a) The wavelength of UVA1 LED with a short wavelength cut‐off filter. (b) Pigmentation was measured in the palm using a spectrophotometric colorimeter (*n* = 6). (c) Summary of the clinical courses of the patients (upper left; *n* = 10). Representative photographs showing cases 1, 2 and 4 before and after a single UVA1 LED treatment. (d) Disease severity was calculated using the Dyshidrotic Eczema Area and Severity Index (DASI) and modified DASI (left, middle; *n* = 10). The duration of acute vesicular stage was shortened (right; *n* = 5). The results were analyzed by Wilcoxon matched‐pairs signed rank test. ns, not significant; **P < 0.01.

In conclusion, the newly developed UVA1 LED irradiation device provided therapeutic effects for dyshidrotic palmoplantar eczema. Compared with local bath‐psoralen and UVA therapy, this UVA LED treatment does not require soaking the palms or use of the photosensitizer 8‐methoxypsoralen. It is also not necessary to avoid sunlight after UVA LED therapy.

## Conflict of Interest

A. M. is the inventor and received a research grant from Ushio. T. K. and H. M. are employees of Ushio. K. I. has no conflict of interest.
